# Synthesis of *Xenia* diterpenoids and related metabolites isolated from marine organisms

**DOI:** 10.3762/bjoc.11.273

**Published:** 2015-12-10

**Authors:** Tatjana Huber, Lara Weisheit, Thomas Magauer

**Affiliations:** 1 Department of Chemistry and Pharmacy, Ludwig-Maximilians-University Munich, Butenandtstraße 5–13, 81377 Munich, Germany

**Keywords:** asymmetric synthesis, natural products, total synthesis, Xenia diterpenoids, xenicanes

## Abstract

This review describes strategies for the chemical synthesis of xenicane diterpenoids and structurally related metabolites. Selected members from the four different subclasses of the *Xenia* diterpenoid family, the xenicins, xeniolides, xeniaphyllanes and xeniaethers, are presented. The synthetic strategies are discussed with an emphasis on the individual key reactions for the construction of the uncommon nine-membered carbocycle which is the characteristic structural feature of these natural products. Additionally, the putative biosynthetic pathway of xenicanes is illustrated.

## Introduction

**Figure 1:**
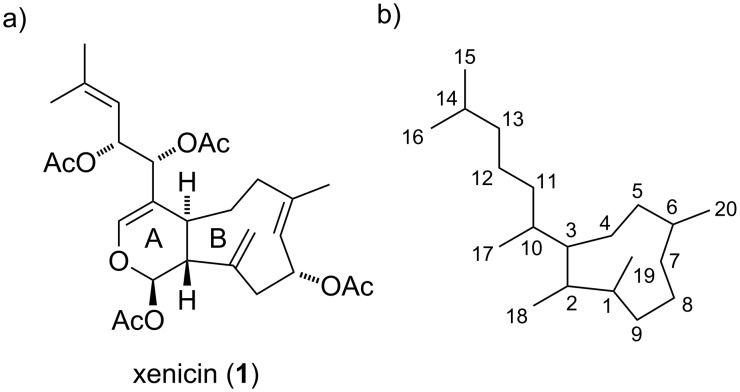
a) Structure of xenicin (**1**) and b) numbering of the xenicane skeleton according to Schmitz and van der Helm.

Terpenoids are a large group of structurally diverse secondary metabolites. Among these natural products, *Xenia* diterpenoids or xenicanes represent a unique family with intriguing structural features and diverse biological activities. Many xenicanes display significant cytotoxic and antibacterial activity and are therefore of great interest for drug discovery, especially for their application as anticancer agents [1]. Marine soft corals of the genus *Xenia* (order *Alcyonacea*, family *Xeniidae*) are known to be rich in xenicane diterpenoids. The first reported member of these metabolites was xenicin (**1**), isolated from the soft coral *Xenia elongata* in Australia, whose structure was elucidated in 1977 by Schmitz and van der Helm (Figure 1a) [2]. The common numbering of the xenicane skeleton shown in Figure 1b is used throughout this review.

**Figure 2:**
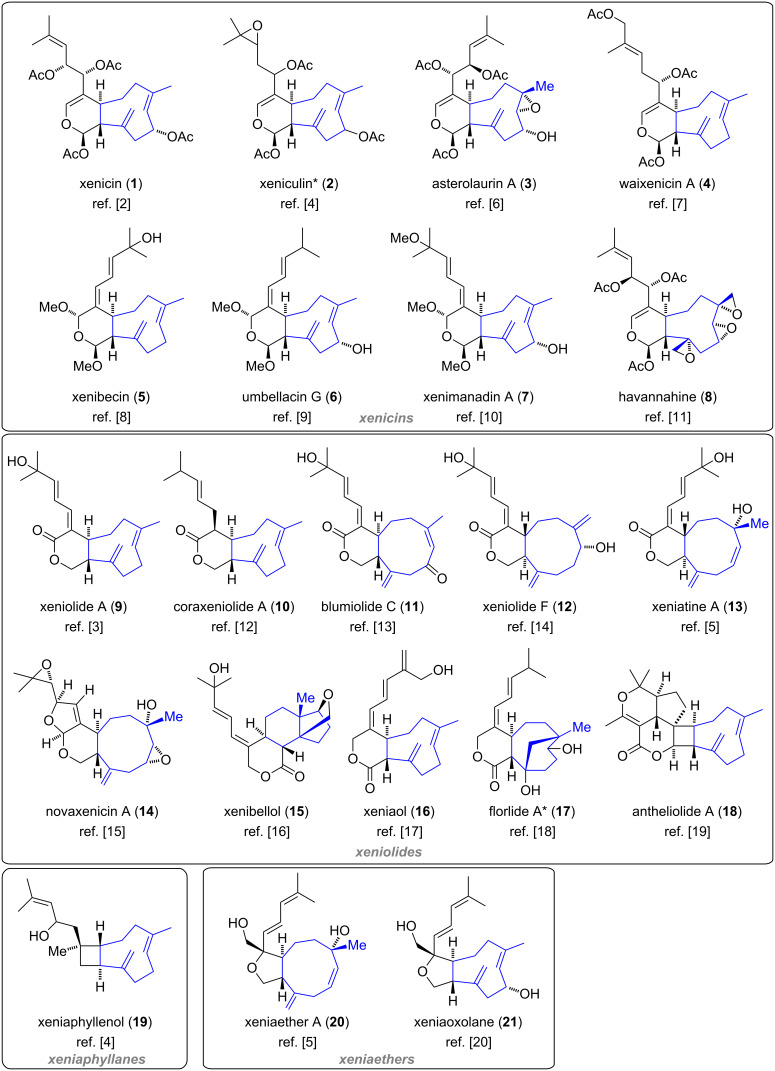
Overview of selected Xenia diterpenoids according to the four subclasses [2–20]. The nine-membered carbocyclic rings are highlighted in blue. *Stereochemistry not determined.

Since then, several further xenicanes with various modifications of the cyclononane ring and isoprenyl side chain in their structure have been isolated. In general, the common structural feature of xenicanes is a bicyclic framework consisting of an A ring which is trans-fused to a nine-membered carbocyclic B ring. The family of *Xenia* diterpenoids was originally divided into three subfamilies: the xenicins (containing an 11-oxabicyclo[7.4.0]tridecane ring system with an acetal functionality) [2], the xeniolides (containing an 11-oxabicyclo[7.4.0]tridecane ring system with a lactone functionality) [3] and the xeniaphyllanes (with a bicyclo[7.2.0]undecane ring system) [4]. Later, an additional subfamily was discovered and named xeniaethers [5] (containing an 11-oxabicyclo[7.3.0]dodecane ring system). An overview of representative members of these subfamilies is depicted in Figure 2.

**Figure 3:**
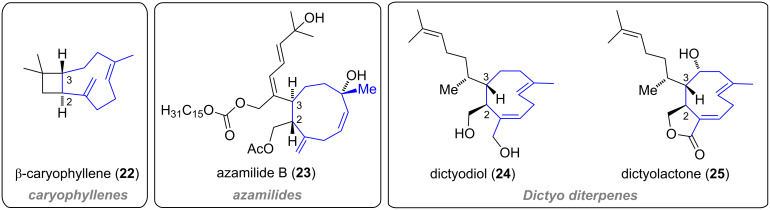
Representative members of the caryophyllenes, azamilides and *Dictyota* diterpenes.

Xenicanes are closely related to a number of metabolites which also feature the characteristic cyclononene framework (Figure 3). For example, a class of bicyclic sesquiterpenes, caryophyllenes [21], exhibit the same bicyclo[7.2.0]undecane skeleton as xeniaphyllanes. Furthermore, while monocyclic azamilides [22] are seco-A-ring diterpenoids that are acylated with fatty acids, *Dictyota* diterpenes [23–24] either bear a similar seco-ring fragment, as observed for dictyodiol (**24**), or comprise a fused γ-butyrolactone moiety, as in dictyolactone (**25**, Figure 3).

This review intends to provide a comprehensive overview of research covering xenicane diterpenoids and related natural products. In the following section, we present a biosynthetic proposal, discuss various synthetic approaches towards xenicane diterpenoids and highlight successful total syntheses.

## Review

### Biosynthetic hypothesis

**Scheme 1:**
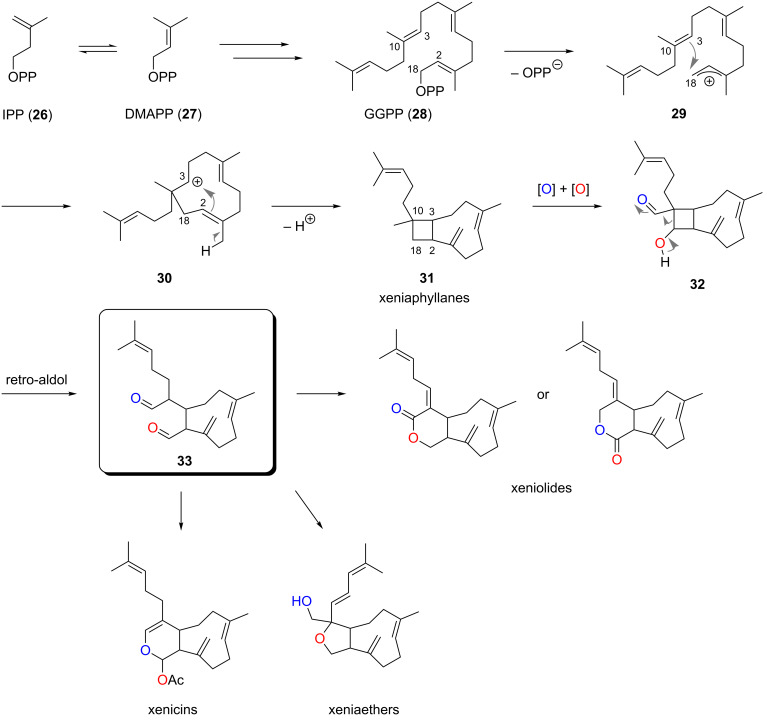
Proposed biosynthesis of *Xenia* diterpenoids (OPP = pyrophosphate, GGPP = geranylgeranyl pyrophosphate, IPP = isoprenyl pyrophosphate, DMAPP = dimethylallyl pyrophosphate).

The proposed biogenesis of xenicanes (Scheme 1) is suggested to be similar to the reported biosynthesis of the structurally related caryophyllene sesquiterpenes [25]. *Xenia* diterpenoids are believed to originate from the common diterpenoid precursor geranylgeranyl pyrophosphate (GGPP, **28**), which is assembled from the two terpene units, isoprenyl pyrophosphate (IPP, **26**) and dimethylallyl pyrophosphate (DMAPP, **27**) [26]. Initial loss of a diphosphate anion from GGPP generates an allylic cation in **29** which is intramolecularly trapped by nucleophilic attack of the C3,C10-double bond, forming the secondary cation **30**. Attack of the newly generated C1,C2-double bond with simultaneous loss of a proton then affords the bicyclo[7.2.0]undecane ring system **31** as found in xeniaphyllanes [3]. Finally, double C–H oxidation furnishes the β-hydroxy aldehyde **32** which can undergo a retro-aldol reaction with concomitant opening of the cyclobutane ring to form dialdehyde **33** as the common biogenetic precursor of xenicins, xeniolides and xeniaethers.

**Scheme 2:**
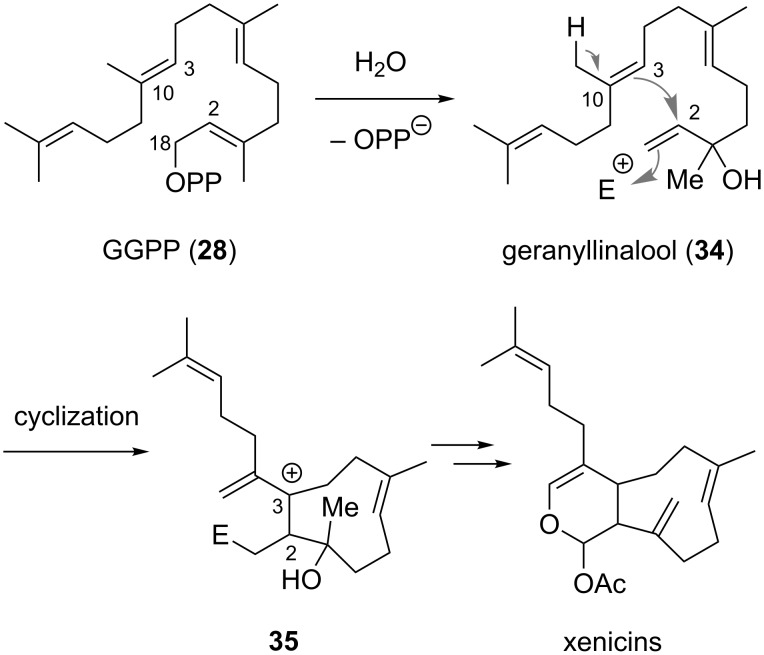
Direct synthesis of the nine-membered carbocycle as proposed by Schmitz and van der Helm (E = electrophilic oxygen species) [2].

An alternative biosynthetic pathway proposed by Schmitz and van der Helm involves the direct formation of the nine-membered carbocyclic ring via oxidative cyclization of geranyllinalool (**34**) [2], which is formed from GGPP (**28**) by enzymatic hydrolysis of the pyrophosphate unit and allylic rearrangement (Scheme 2).

### Synthetic strategies

The unusual molecular structures and the potential of xenicanes to act as chemotherapeutic agents make these natural products attractive targets for synthetic chemists. Although more than 100 different *Xenia* diterpenoids are known to date, only a few total syntheses of xeniolides have been reported in the last two decades. Surprisingly, since the discovery of xenicin in 1977 [2], no total synthesis of a member of this subclass has been accomplished.

**Scheme 3:**
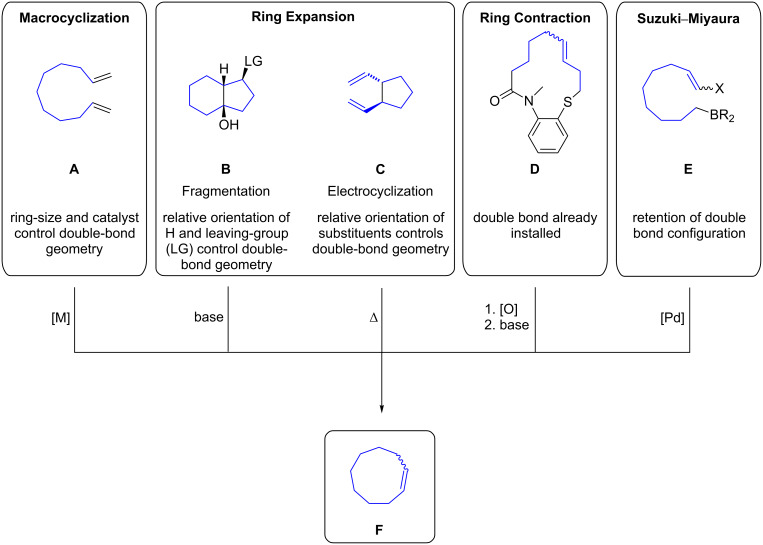
The construction of *E-* or *Z-*cyclononenes.

The synthesis of nine-membered rings is challenging, especially when they contain an *E*-configured double bond. Different strategies for the construction of *E-* or *Z-*cyclononenes have been reported to date and common reactions are summarized in Scheme 3. Transition metal-catalyzed ([M] = Ru, Mo, W) ring-closing metathesis (RCM) reactions of 1,10-dienes **A** can be employed for the synthesis of cyclononenes. The *E*/*Z*-selectivity of the olefin depends on the ring-size and the choice of catalyst. As a consequence of avoiding ring strain, small- and medium-sized rings are generally obtained with *Z*-configuration of the alkene. The Grob fragmentation reaction of fused 6,5-bicycles **B** is usually a concerted process that affords cyclononenes in a stereospecific manner [27]. The relative configuration of the leaving group (LG = OTs, OMs, Hal, NR_3_^+^) and the adjacent substituent determine the *E*/*Z*-geometry of the olefin. A *cis*-geometry leads to the formation of the *E*-configured double bond. In general, the Grob fragmentation is the most commonly employed method for the synthesis of cyclononenes due to the predictability of the stereochemical outcome of the product. The construction of cyclononenes can furthermore be achieved by thermal [3,3]-sigmatropic rearrangements of 1,5-dienes **C**. When the reaction proceeds via a chairlike transition state, the substituents are oriented with minimal steric hindrance to give the *E*,*E*-configured nine-membered ring. Ring contraction reactions of 13-membered lactams afford cyclononenes via intramolecular acyl transfer reactions. The configuration of the double bond derives from precursor **D** and thus allows the formation of *E*- or *Z*-configured cyclononenes. Additionally, the intramolecular palladium-catalyzed cyclization of haloalkenes with organoboranes affords cyclononenes with retention of the double bond configuration [28]. The corresponding allylic alcohols can be prepared by a Nozaki–Hiyama–Kishi coupling of haloalkenes with aldehydes.

**Scheme 4:**
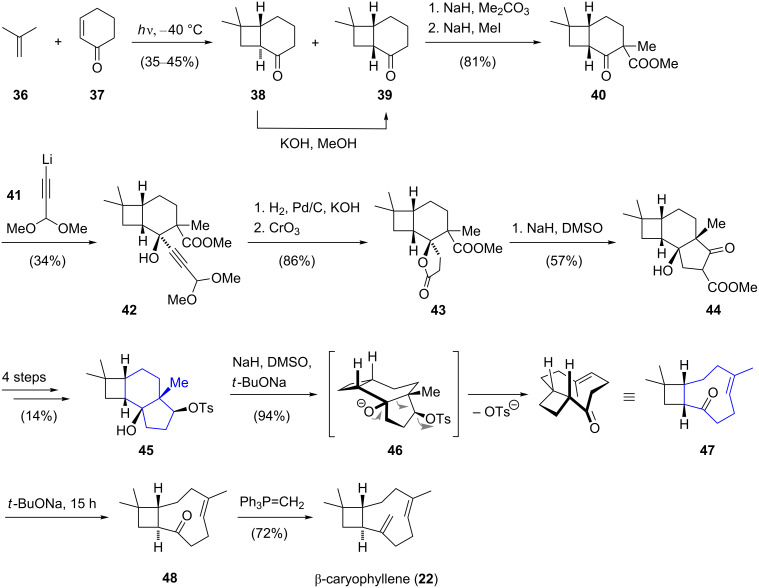
Total synthesis of racemic β-caryophyllene (**22**) by Corey.

The first synthesis of the unusual nine-membered carbocyclic ring was reported by Corey for the total synthesis of β-caryophyllene in 1963 (Scheme 4) [29–31]. Starting with a photochemical [2 + 2] cycloaddition between 2-cyclohexen-1-one (**37**) and isobutene (**36**), an isomeric mixture of *trans*- and *cis*-fused [4.2.0]octanone was obtained (*trans*-**38**/*cis*-**39** = 4:1). The more stable *cis*-bicycle **39** could be obtained by isomerization of *trans-***38** with base. Acylation with sodium hydride and dimethyl carbonate followed by methylation furnished β-keto ester **40**. Addition of lithium acetylide **41** to the keto group led to acetal **42**. Hydrogenation of the triple bond under basic conditions resulted in cleavage of the acetal and ring closure to the corresponding lactol which was oxidized with chromic acid to furnish γ-lactone **43**. An ensuing Dieckmann condensation [32] of **43** afforded a 4,6,5-tricycle which was converted to the fragmentation precursor **45** in four further steps. A base-mediated Wharton-type Grob fragmentation [33] then served as the key step to construct the cyclononene motif of bicycle **47**. Prolonged exposure of the resulting *cis*-fused 4,9-bicycle **47** to sodium *tert*-butoxide gave rise to the epimerized *trans*-isomer **48**. Finally, the exocyclic double bond was introduced by olefination of ketone **48** and thus completed the racemic total synthesis of β-caryophyllene (**22**) in 13 steps. This elegant synthesis received considerable attention and revealed already at that time the great potential of modern synthetic organic chemistry.

**Scheme 5:**
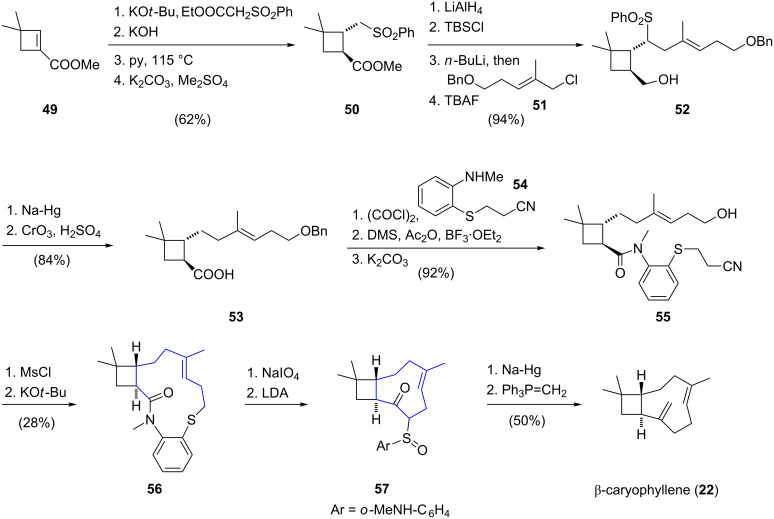
Total synthesis of racemic β-caryophyllene (**22**) by Oishi.

More than 20 years later, in 1984, Oishi and co-workers reported a different strategy which culminated in the total synthesis of racemic β-caryophyllene (**22**) (Scheme 5) [34]. Their synthesis commenced with conjugate addition of ethyl (phenylsulfonyl)acetate, a methylsulfonyl anion equivalent, to cyclobutene ester **49** followed by a sequence consisting of saponification, regioselective decarboxylation and reesterification to afford methyl ester **50**. The ester group was reduced with lithium aluminum hydride and the resulting alcohol was converted to the corresponding silyl ether. Next, alkylation of the metalated sulfone with allylic chloride **51** afforded alcohol **52** after desilylation. Subsequent desulfonylation with sodium amalgam and Jones oxidation of the primary alcohol furnished carboxylic acid **53**. The corresponding tertiary amide was then formed by sequential reaction of carboxylic acid **53** with oxalyl chloride and *N*-methylaniline derivative **54**. The following two-step debenzylation sequence afforded alcohol **55** which was converted to the corresponding mesylate, serving as a key intermediate for the construction of the nine-membered carbocyclic ring. Treatment of this intermediate with potassium *tert*-butoxide led to the cleavage of the 2-cyanoethylsulfide moiety and the generation of a thiolate anion, which underwent S_N_2 displacement of the primary mesylate, affording the 13-membered lactam **56**. The stage was now set for the key intramolecular acyl transfer reaction to form the cyclononene motif. After sodium periodate oxidation of sulfide **56** to the corresponding sulfoxide, addition of lithium diisopropylamide initiated the intramolecular acyl transfer and led to formation of cyclononene **57** in quantitative yield. Reductive desulfonylation and a final Wittig olefination of the ketone then afforded racemic β-caryophyllene (**22**). In summary, the total synthesis of β-caryophyllene was achieved in 19 steps with an overall yield of 6.3%. Although the key intramolecular acyl transfer reaction for construction of the cyclononene ring could be realized in quantitative yield, the low-yielding formation of the macrocyclic thioether reduced the overall efficiency of the presented synthetic route. Based on a similar strategy and using the corresponding *Z*-isomer of cyclization precursor **39**, Oishi and co-workers reported a total synthesis of racemic isocaryophyllene, the *cis* double bond isomer of caryophyllene. Further total syntheses of isocaryophyllene have also been reported by Kumar [35–36], Miller [37] and Bertrand [38].

**Scheme 6:**
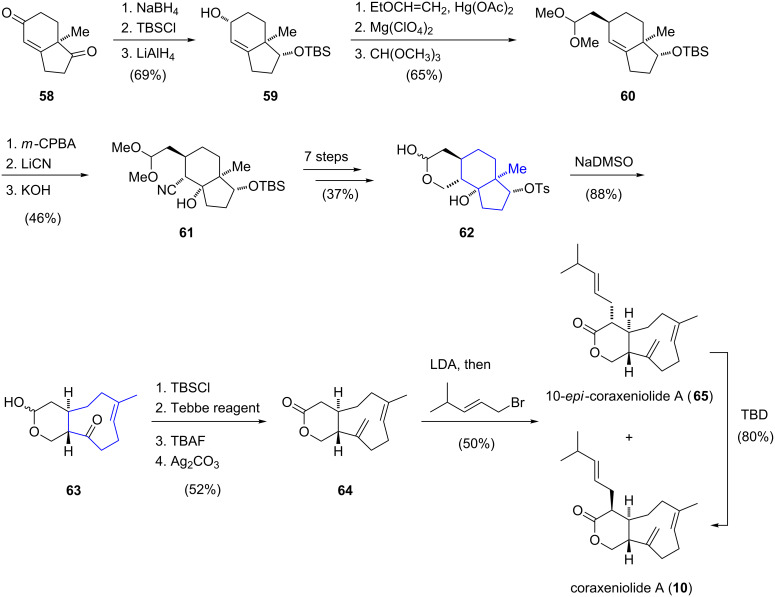
Total synthesis of coraxeniolide A (**10**) by Leumann.

In 1995, Pfander reported the synthesis of an important building block [24] for the total synthesis of coraxeniolide A (**10**) [12], starting from chiral (−)-Hajos–Parrish diketone (**58**) [39]. Based on Pfander's seminal work, the first total synthesis of a xenicane diterpenoid was then accomplished by Leumann in 2000 (Scheme 6) [40]. Starting from enantiopure (−)-Hajos–Parrish diketone (**58**), allylic alcohol **59** was prepared by regioselective reduction of the carbonyl group, silylation of the resulting alcohol and further reduction of the enone moiety. An ensuing transetherification of alcohol **59** with ethyl vinyl ether gave an allyl vinyl ether, which underwent a magnesium perchlorate-promoted [1,3]-sigmatropic rearrangement [41] to afford an aldehyde that was converted to dimethylacetal **60**. The following epoxidation proceeded with good stereoselectivity (α/β ≈ 11:1) and the regioselective opening of the epoxide moiety using lithium cyanide afforded a β-hydroxy nitrile in a *trans*-diaxial arrangement. Under basic conditions, the configuration of the nitrile group at C2 was inverted, furnishing the thermodynamically more stable **61**. Nitrile **61** was then converted to lactol **62** in seven further steps. Next, the cyclononene ring of **63** was constructed via a Grob fragmentation of 6,6,5-tricycle **62**, affording the bicyclic product **63** in very good yield, however, as a mixture of lactol epimers (α/β ≈ 56:44). Silyl protection of the lactol and subsequent Tebbe olefination [42] of the ketone group installed the exocyclic double bond of the nine-membered carbocycle. Desilylation followed by oxidation with silver carbonate then afforded lactone **64**. For the introduction of the side chain, the enolate derived from lactone **64** was treated with 1-bromo-4-methylpent-2-ene, giving a 1:6 mixture of coraxeniolide A (**10**) and its epimer **65**. By equilibration with triazabicyclodecene (TBD), the ratio of **10**:**65** could be inverted to 3:1. In summary, coraxeniolide A (**10**) was synthesized in a longest linear sequence of 23 steps with an overall yield of 1.4%.

**Scheme 7:**
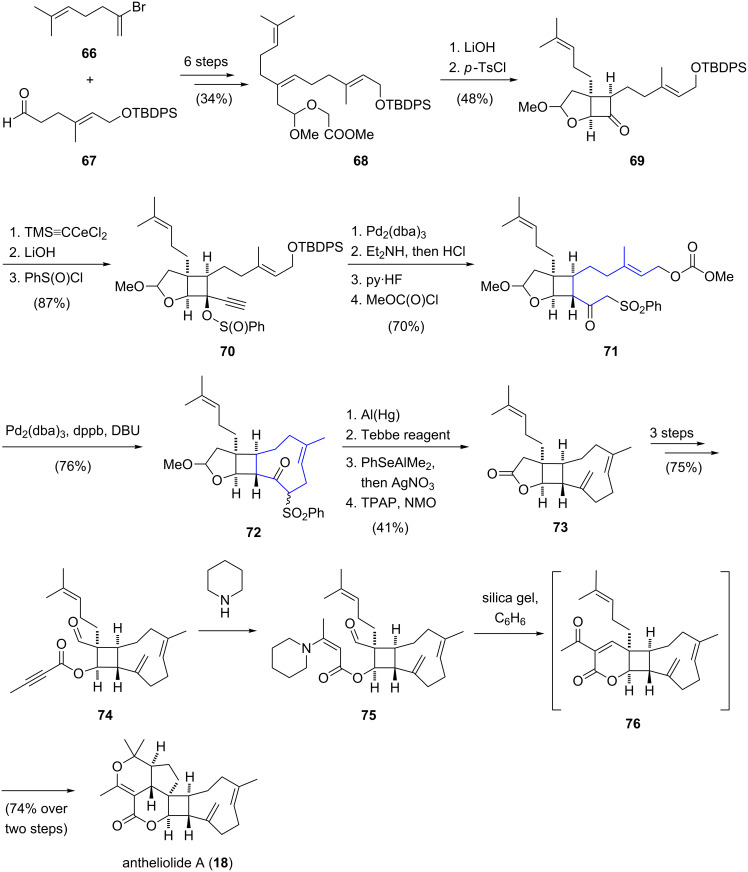
Total synthesis of antheliolide A (**18**) by Corey.

The most complex xenicane diterpenoid synthesized to date is pentacyclic antheliolide A (**18**) [18] by Corey (Scheme 7) [43]. The linear precursor **68** was prepared from vinyl bromide **66** and aldehyde **67** in six steps in 34% yield. After saponification of the ester functionality, treatment with tosyl chloride and trimethylamine resulted in the formation of a ketene that underwent a diastereoselective intramolecular [2 + 2] cycloaddition to provide bicyclic ketone **69**. Addition of TMS cerium acetylide to the carbonyl group of **69**, followed by desilylation under basic conditions gave rise to (±)-ethynylcarbinol, which was separated by chiral HPLC. The desired diastereomer was then transformed to benzene sulfinate ester **70**. A palladium-catalyzed [2,3]-sigmatropic rearrangement formed an isomeric allenic sulfone [44] which, upon conjugate addition of diethyl amine followed by hydrolysis afforded a β-ketosulfone. For the following ring closure, the primary alcohol was desilylated and converted to the corresponding allylic carbonate **71**. The cyclononene structure **72** was then assembled via a palladium-catalyzed and base-mediated cyclization of carbonate **71** [45]. Reductive cleavage of the sulfone using aluminium amalgam afforded a ketone, which was converted to an exocyclic double bond by treatment with Tebbe’s reagent [42]. In order to convert the methoxy acetal to the corresponding lactone, without affecting the sensitive caryophyllene-like subunit, the methoxy group was replaced with a phenylseleno moiety, which was converted to the alcohol and finally oxidized to lactone **73**. In three further steps, lactone **73** was converted to aldehyde ester **74**, which upon treatment with piperidine gave a β-enamino ester **75**. Finally, an elegant cascade reaction involving an aldol condensation, followed by a hetereo Diels–Alder reaction closed the last three rings and antheliolide A (**18**) was obtained in 74% yield. In summary, the successful total synthesis of antheliolide A proceeded in 25 linear steps with an overall yield of 1.7%.

**Scheme 8:**
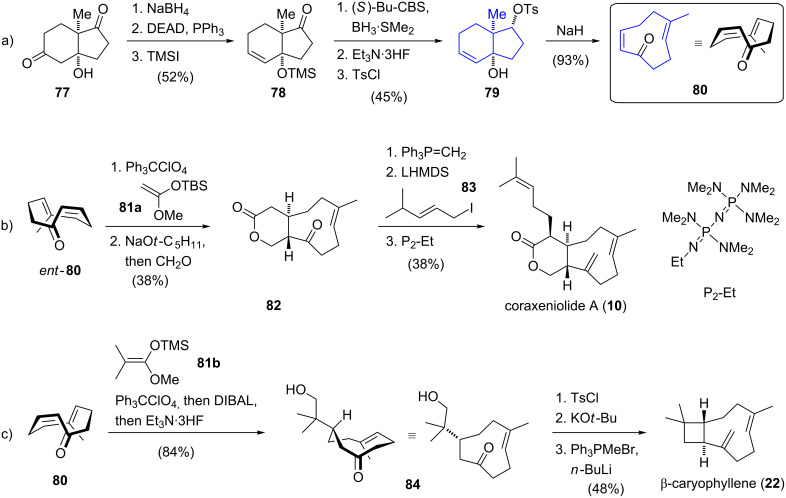
a) Synthesis of enantiomer **80**, b) total syntheses of coraxeniolide A (**10**) and c) β-caryophyllene (**22**) by Corey.

The total syntheses of coraxeniolide A (**10**) and β-caryophyllene (**22**) reported by Corey [46] in 2008 are based on Pfander’s idea [24] to construct the cyclononene fragment from (−)-Hajos–Parrish diketone (**58**) [39] (Scheme 8). Chiral hydroxy dione **77** was synthesized according to a literature-known procedure [47]. Regioselective reduction with sodium borohydride, followed by dehydration under Mitsunobu conditions and silylation of the tertiary alcohol furnished trimethylsiloxy ketone **78**. The ketone functionality was then diastereoselectively reduced under Corey–Bakshi–Shibata conditions [48] and an ensuing desilylation furnished a diol. In order to introduce a leaving group for the following key step, the secondary hydroxy group was tosylated to afford **79**. Once again, a stereospecific Grob fragmentation of tosylate **79** served as the key step for the synthesis of the enantiomerically pure and configurationally stable nine-membered *E*,*Z*-dienone **80**. The synthesis of the enantiomer of dienone **80**, *ent*-**80**, was accomplished by a route parallel to that presented in Scheme 8a, starting from *ent*-**77**. The highly efficient construction of these versatile intermediates provides a basis to synthesize a variety of natural products containing this macrocyclic structural motif. Based on chiral enone **80** and its enantiomer, *ent*-**80**, coraxeniolide A (**10**) and β-caryophyllene (**22**) were synthesized in five and four further steps, respectively. The synthesis of **10** continued with a trityl perchlorate-catalyzed conjugate addition of silyl ketene acetal **81a** to enone *ent*-**80**. Deprotonation and trapping of the resulting enolate with formaldehyde furnished lactone **82** in a regio- and stereoselective fashion. Introduction of the exocyclic double bond proved to be challenging and therefore salt-free, highly reactive methylenetriphenylphosphorane was used. Finally, α-alkylation of the lactone with iodide **83** provided coraxeniolide A (**10**) and its epimer in a 1:6 ratio which could be reversed to 4:1 by base-mediated equilibration. Purification by column chromatography, allowed the two epimers to be separated and afforded coraxeniolide A (**10**) in 38% yield over three steps.

Additionally, the enantioselective total synthesis of β-caryophyllene was realized starting from key intermediate **80**. The route commenced with conjugate addition of silyl ketene acetal **81b** to enone **80** from the sterically less hindered re-face. The ester group was selectively reduced and desilylation afforded alcohol **84**. The generated primary alcohol was tosylated and regioselective deprotonation followed by intramolecular α-alkylation stereoselectively formed the cyclobutane ring. A final Wittig methylenation introduced the exocyclic double bond and afforded (−)-β-caryophyllene (**22**), for the first time in an enantioselective manner. In conclusion, Corey's protocol for the synthesis of a highly versatile building block represents a valuable platform for the construction of many different metabolites containing the nine-membered carbocyclic ring segment. The application of this useful intermediate was elegantly demonstrated in the synthesis of coraxeniolide A proceeding in 14% yield over five steps.

**Scheme 9:**
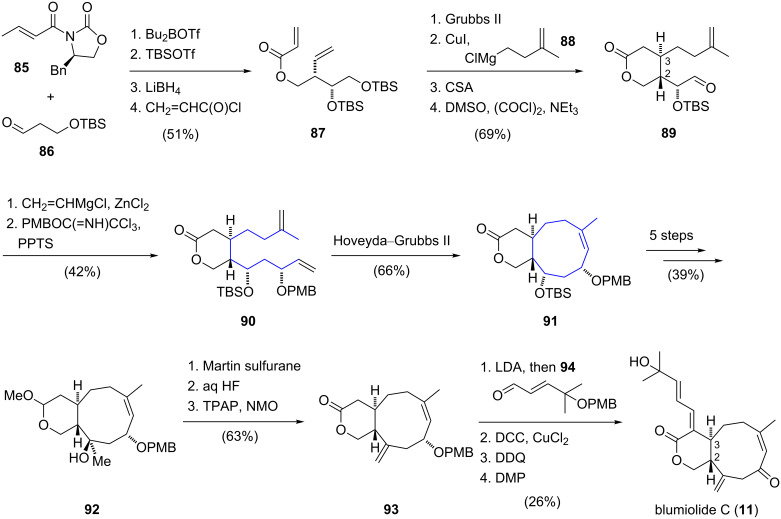
Total synthesis of blumiolide C (**11**) by Altmann.

Altmann and co-workers disclosed the total synthesis of blumiolide C (**11**) [20] employing a *Z*-selective ring-closing metathesis reaction for construction of the cyclononene unit [49]. The synthesis started with a diastereoselective Evans syn-aldol reaction between substituted propanal **86** and *E*-crotonyl-oxazolidinone **85** (Scheme 9). The resulting secondary alcohol was silylated and the chiral auxiliary was cleaved with lithium borohydride. Acylation with acryloyl chloride gave ester **87** and a ring-closing metathesis reaction using Grubbs second generation catalyst [50] furnished an α,β-unsaturated lactone. Subsequent 1,4-addition of the cuprate derived from alkylmagnesium chloride **88** provided the *trans*-product with excellent diastereoselectivity and thus installed the required stereocenter at the C3 position of the natural product. After deprotection of the sterically less hindered silyl ether, the resultant primary alcohol was oxidized to give aldehyde **89**. By treatment with in situ generated divinylzinc, aldehyde **89** was transformed to an allylic alcohol which was converted to the corresponding *para*-methoxybenzyl ether **90** using Bundle's reagent [51]. In the key step of the synthesis, the nine-membered carbocyclic ring was constructed via a ring-closing metathesis reaction. Under optimized conditions, Hoveyda–Grubbs second generation catalyst [52] selectively converted diene **90** to the bicyclic ring system **91** in 66% yield. For the installation of the exocyclic double bond, bicycle **92** was treated with Martin sulfurane [53]. Subsequent hydrolysis of the acetal functionality and oxidation of the resulting lactol restored the lactone function in bicycle **93**. The side chain of blumiolide C was introduced by an aldol reaction between lactone **93** and aldehyde **94**. In the final sequence, blumiolide C (**11**) was obtained via stereospecific dehydration, removal of the *para*-methoxybenzyl ether and oxidation. In summary, the total synthesis of blumiolide C was accomplished in an overall yield of 0.63%.

**Scheme 10:**
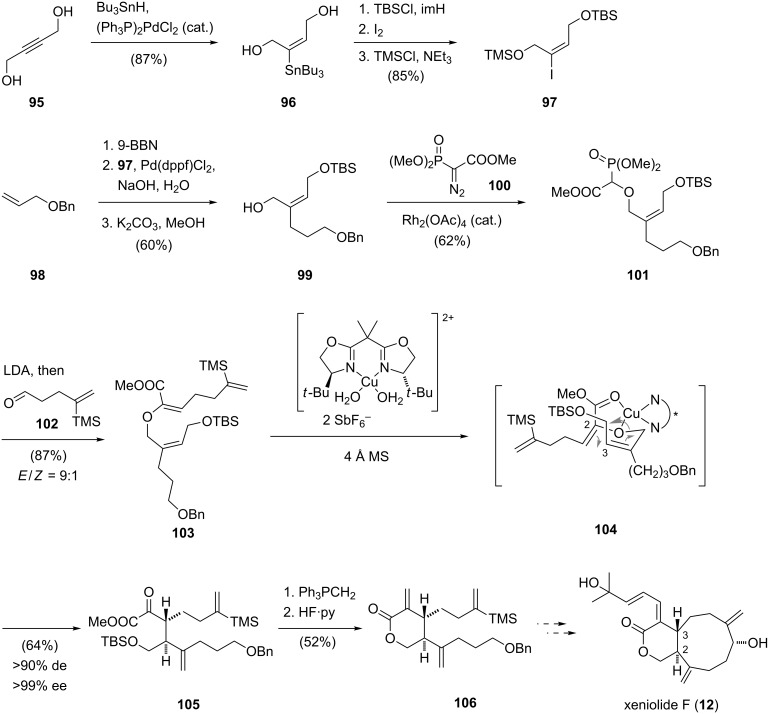
Synthesis of a xeniolide F precursor by Hiersemann.

In 2005, Hiersemann and co-workers reported an approach towards the synthesis of xeniolide F [13] employing a catalytic asymmetric Claisen rearrangement to set the crucial stereocenters at the C2 and C3 positions (Scheme 10) [54]. The synthesis commenced with the preparation of diol **96** by a palladium-catalyzed hydrostannylation of 2-butyne-1,4-diol (**95**). Regioselective silylation with *tert*-butyldimethylsilyl chloride of the sterically less hindered alcohol, iodination and silylation of the primary alcohol with trimethylsilyl chloride gave vinyl iodide **97**. The following palladium-catalyzed B-alkyl Suzuki–Miyaura cross coupling between the borane derived from alkene **98** and vinyl iodide **97** furnished a *Z*-configured alkene. Deprotection of the trimethylsilyl ether then afforded alcohol **99**. A rhodium(II)-catalyzed O–H insertion reaction of the rhodium carbenoid derived from diazophosphonoacetate **100** and alcohol **99** afforded intermediate **101** which was treated with lithium diisopropylamide and aldehyde **102** to afford alkene **103** with high *E*-selectivity. The following asymmetric copper(II)-catalyzed Claisen rearrangement [55], which is postulated to proceed via the chair-like transition state **104**, afforded key intermediate **105** with high diastereo- and enantioselectivity. Preparation of the δ-lactone **106** of the A ring of xeniolide F was then realized by treatment of Claisen product **105** with the methylene Wittig reagent, followed by desilylation and lactonization. Although a successful synthetic approach leading to lactone **106** was thus established, further efforts to complete the total synthesis of xeniolide F (**12**) have yet to be reported.

**Scheme 11:**
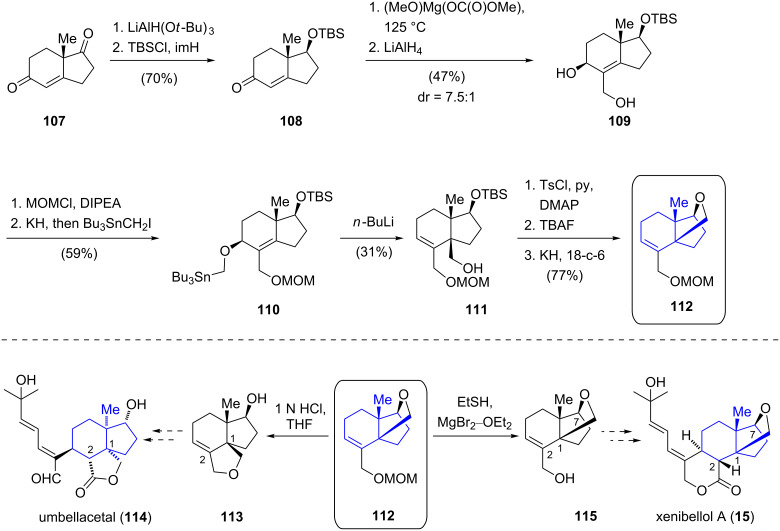
Synthesis of the xenibellol (**15**) and the umbellacetal (**114**) core by Danishefsky.

Efforts aimed at constructing the core structure of xenibellol A (**15**) [15] and umbellacetal (**114**) [56] employing a 2,3-Wittig–Still rearrangement as the key step were reported by Danishefsky and co-workers (Scheme 11) [57]. In contrast to other xenicanes mentioned above, xenibellol A (**15**) does not possess the characteristic nine-membered carbocyclic ring but rather features a 6,5,5-ring system, containing an unusual oxolane bridge between C1 and C7. Hajos–Parrish diketone (**107**) [39] served as the starting material for the preparation of key intermediate **112**. Selective reduction of the ketone and silylation of the resulting alcohol furnished enone **108**. α-Carboxylation of the enone with magnesium methyl carbonate and a global reduction of the carbonyl functionalities afforded allylic alcohol **109**. The precursor for the key reaction was obtained by formation of the methoxymethyl (MOM) ether from primary alcohol **109** and subsequent conversion of the allylic alcohol to stannane **110**. The following 2,3-Wittig–Still rearrangement [58] employing *n*-butyllithium afforded primary alcohol **111** in 31% yield and enabled the installation of the C1 quaternary stereocenter. According to the authors, a competing 1,2-Wittig rearrangement and reduction pathway posed a significant challenge in this transformation. Desilylation and regioselective tosylation of the primary alcohol **111** set the stage for the construction of the oxolane via Williamson etherification, which was realized by treatment with potassium hydride. Surprisingly, the following deprotection of the MOM ether using standard reaction conditions (1 N aqueous hydrochloric acid) led to opening of the oxolane ring and afforded tricycle **113** which features the carbon framework of structurally related umbellacetal (**114**). Gratifyingly, when magnesium bromide and ethanethiol were used as a mild alternative for the cleavage of the MOM ether, the xenibellol core could be obtained. Although the key 2,3-Wittig–Still rearrangement proceeded in low yield and further improvements are necessary, a promising route towards the synthesis of umbellacetal (**114**) and xenibellol (**15**) was thus established.

**Scheme 12:**
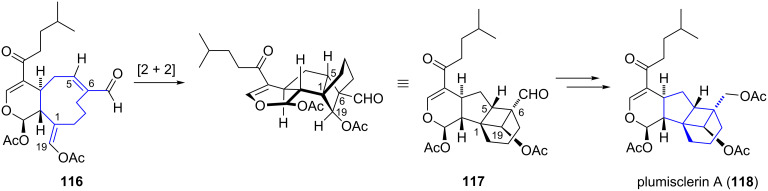
Proposed biosynthesis of plumisclerin A (**118**).

Yao and co-workers have investigated a synthetic approach towards the soft coral metabolite plumisclerin A by Pauson–Khand annulation and SmI_2_-mediated radical cyclization [59]. The xenicane-related diterpenoid (isolated from the same marine organism as xenicin **116**) possesses a complex ring system that is proposed to be biosynthetically derived from the xenicin diterpenoid **116** by an intramolecular [2 + 2] cycloaddition (Scheme 12) [60].

**Scheme 13:**
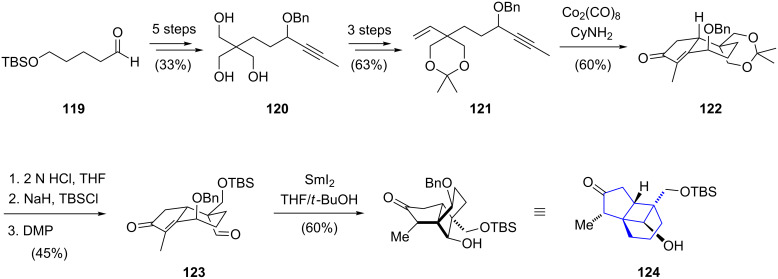
Synthesis of the tricyclic core structure of plumisclerin A by Yao.

The synthetic route commenced with known aldehyde **119** which was converted to triol **120** in five steps (Scheme 13). The introduction of the benzyl ether next to the alkyne moiety was necessary to control the stereochemical outcome of the key annulation, and further three steps enabled preparation of the annulation precursor **121**. The following Pauson–Khand reaction [61] for the construction of the fused bicyclic structure **122** was performed by treatment of **121** with dicobaltoctacarbonyl in the presence of cyclohexylamine. Hydrolysis of the acetonide, chemoselective silylation and oxidation afforded aldehyde **123**. Next, the formation of the cyclobutanol ring was realized by an intramolecular samarium diiodide-mediated radical conjugate addition to afford tricycle **124** in 60% yield. Introduction of the dihydropyran ring of plumisclerin A (**118**) was envisioned to be carried out at a late stage of the synthesis, but efforts towards its construction have yet to be reported.

**Scheme 14:**
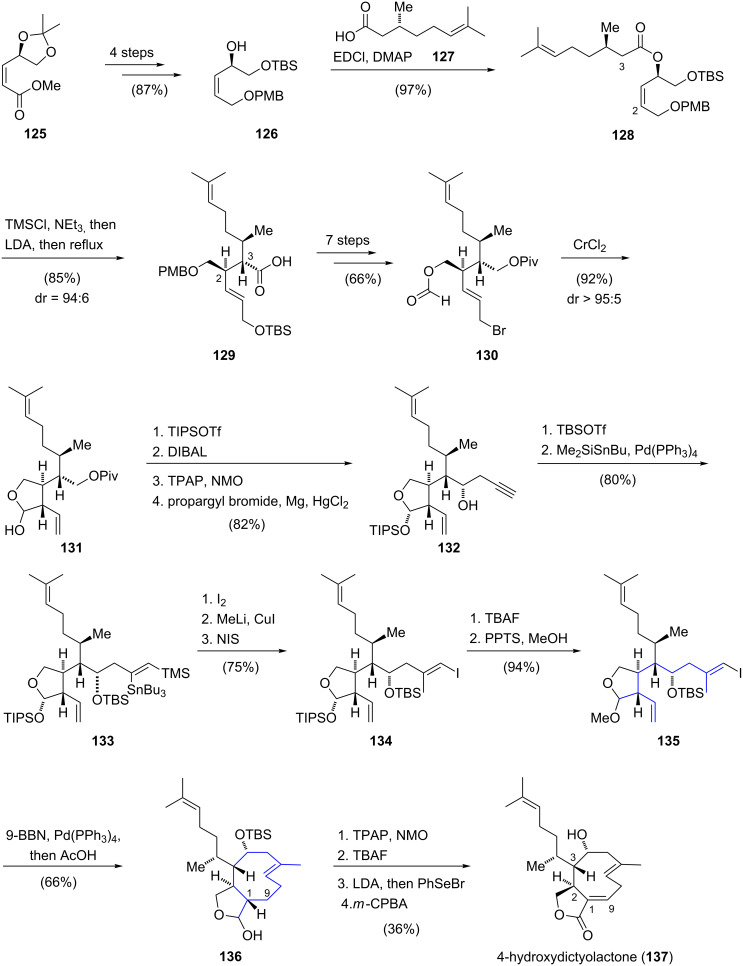
Total synthesis of 4-hydroxydictyolactone (**137**) by Williams.

In 2009, the enantioselective total synthesis of 4-hydroxydictyolactone (**137**) was reported by Williams and co-workers (Scheme 14) [62]. Starting from α,β-unsaturated ester **125**, allylic alcohol **126** was synthesized in four steps. Esterification with (*R*)-(+)-citronellic acid (**127**) yielded a single diastereomer of ester **128**. Addition of lithium diisopropylamide to a mixture of **128**, trimethylsilyl chloride and triethylamine initiated an Ireland–Claisen rearrangement [63] which gave carboxylic acid **129** in 85% yield and with high diastereoselectivity (dr = 94:6). Carboxylic acid **129** was then converted to intermediate **130** in seven further steps. An intramolecular coupling between the formate ester and the allylic bromide provided lactol **131** in excellent stereoselectivity (dr > 95:5). The preparation of secondary alcohol **132** was accomplished by cleavage of the pivaloate ester, oxidation under Ley–Griffith oxidation [64] and subsequent addition of propargylmagnesium bromide. *O*-Silylation of the propargylic alcohol followed by a regioselective palladium-catalyzed *syn*-silylstannylation yielded product **133**. After employing a three-step protocol for the sequential replacement of the stannyl and silyl substituents, *E*-vinyl iodide **134** was obtained with retention of the olefin geometry. The following intramolecular key coupling step between the vinyl iodide and the terminal alkene for the formation of the nine-membered carbocycle was realized via a B-alkyl Suzuki–Miyaura cross-coupling reaction. Optimization studies of this key ring closure with different protecting groups on the lactol functionality revealed methyl acetal **135** as the most efficient substrate for this transformation. The challenging key step was finally realized in 66% yield and gave, after hydrolysis of the acetal with acetic acid, a mixture of *trans*-fused diastereomers **136**. Finally, a sequence consisting of oxidation, deprotection of the silyl ether and selenoxide elimination introduced the C1,C9 double bond to furnish 4-hydroxydictyolactone (**137**). In summary, the total synthesis of 4-hydroxydictyolactone was successfully completed in 30 linear steps with an overall yield of 4.8%.

**Scheme 15:**
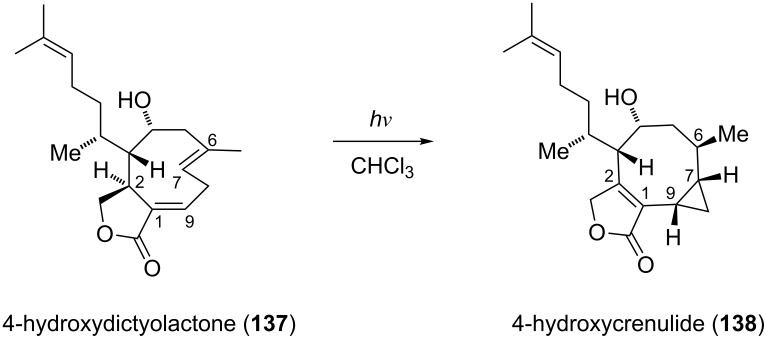
Photoisomerization of 4-hydroxydictyolactone (**137**) to 4-hydroxycrenulide (**138**).

Paquette and co-workers disclosed the enantioselective total synthesis of the *Xenia* diterpenoid related crenulatane (+)-acetoxycrenulide (**151**) [65–67]. The skeleton of crenulatanes, which features an eight-membered carbocyclic ring fused to a cyclopropane ring, may be the product of a photoisomerization of xenicanes. This hypothesis was further supported by the fact that crenulatanes usually co-occur with xenicanes in brown seaweeds of the family *Dictyotaceae*. Evidence for this proposed biogenetic origin of crenulatanes has been provided by Guella and Pietra who showed that irradiation of 4-hydroxydictyolactone (**137**) with ultraviolet light (254 nm) led to the formation of 4-hydroxycrenulide (**138**) (Scheme 15) [68]. Although this transformation remains mechanistically unclear, the authors suggested that either a free radical process or a photoinduced double bond isomerization (C9,C1 to C1,C2) followed by an [1,3]H shift might lead to the formation of 4-hydroxycrenulide (**138**).

**Scheme 16:**
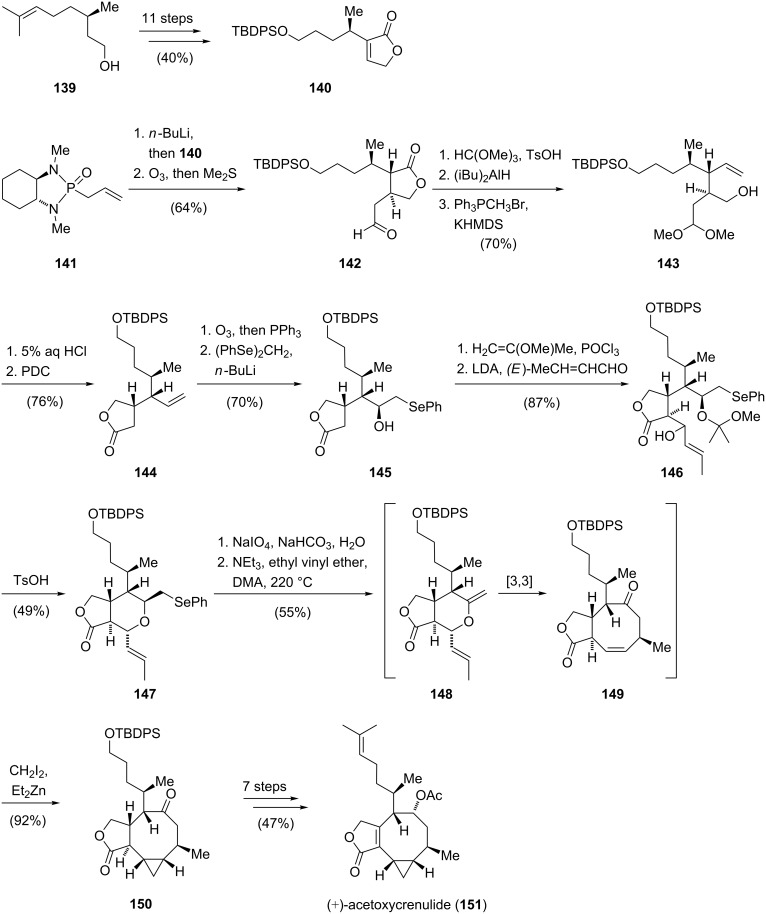
The total synthesis of (+)-acetoxycrenulide (**151**) by Paquette.

The total synthesis of (+)-acetoxycrenulide (**151**) commenced with preparation of butenolide **140** from (*R*)-citronellol (**139**) in an 11-step sequence. Next, the two stereocenters at C2 and C3 position were installed by stereoselective conjugate addition of enantiopure α-allylphosphonamide **141** to butenolide **140**. After cleavage of the chiral auxiliary by ozonolysis, aldehyde **142** was protected as the dimethoxy acetal and reduction of the lactone followed by olefination furnished alkene **143**. The lactone fragment of the natural product was then installed by acidic hydrolysis of the acetal functionality and subsequent oxidation gave γ-lactone **144**. Ozonolysis of the terminal alkene and addition of (phenylseleno)methyllithium to the resulting aldehyde afforded secondary alcohol **145**. Temporary protection of the alcohol followed by an aldol reaction of the lactone with *E*-crotonaldehyde led to an inseparable mixture (dr = 1:1) of β-hydroxy lactone **146**. The synthesis of the key precursor for formation of the cyclooctene core was achieved via an acid-catalyzed cyclization to form tetrahydropyran **147**. The following key sequence consisted of a thermal selenoxide 1,2-elimination to generate allyl vinyl ether **148** which underwent a stereoselective Claisen rearrangement [69] to furnish cyclooctenone **149** in 55% yield. A highly stereoselective Simmons–Smith reaction [70] delivered the cyclopropyl ring exclusively from the accessible α-face to give **150**. The synthesis of (+)-acetoxycrenulide (**151**) was completed in seven further steps and in summary proceeded in 33 steps (longest linear sequence) and in 1% overall yield (Scheme 16).

In addition to the presented strategies for the synthesis of *Xenia* diterpenoids, total syntheses of the *Xenia* sesquiterpenes xenitorin B and C were also reported [71].

## Conclusion

This review has presented various synthetic approaches towards xenicane and xenicane-related diterpenoids. Additionally, total syntheses of xeniolides and of a crenulatane natural product were illustrated. It has been shown that the rare structural features of *Xenia* diterpenoids represent an enduring challenge for the total synthesis of these fascinating metabolites. For these reasons, several strategies for the preparation of the characteristic nine-membered carbocyclic ring structures have been developed. The synthetic strategies are typically based on ring expansion (Grob-type fragmentation and sigmatropic rearrangements), ring closing (metathesis and transition metal-catalyzed coupling) and ring contracting reactions. The choice of tactic is dependent on the individual substitution pattern of the target compound. However, many of the presented strategies rely on long synthetic sequences that cannot provide large amounts of synthetic material which is required for further investigations of the biological activity of these natural products, and ultimately for drug discovery. The development of short and efficient synthetic routes towards xenicane natural products therefore remains a great challenge of this exciting research field.
